# Understanding low-value care and associated de-implementation processes: a qualitative study of Choosing Wisely Interventions across Canadian hospitals

**DOI:** 10.1186/s12913-022-07485-6

**Published:** 2022-01-21

**Authors:** Gillian Parker, Monika Kastner, Karen Born, Nida Shahid, Whitney Berta

**Affiliations:** 1 Institute of Health Policy, Management and Evaluation, University of Toronto, 155 College Street, 4th Floor, Toronto, ON M5T 3M6 Canada; 2 North York General Hospital, Centre for Research and Innovation, 4001, Leslie Street, Toronto, ON M2K 1E1 Canada

**Keywords:** Choosing Wisely, De-implementation, Low-value care, Qualitative

## Abstract

**Background:**

Choosing Wisely (CW) is an international movement comprised of campaigns in more than 20 countries to reduce low-value care (LVC). De-implementation, the reduction or removal of a healthcare practice that offers little to no benefit or causes harm, is an emerging field of research. Little is known about the factors which (i) sustain LVC; and (ii) the magnitude of the problem of LVC. In addition, little is known about the processes of de-implementation, and if and how these processes differ from implementation endeavours. The objective of this study was to explicate the myriad factors which impact the processes and outcomes of de-implementation initiatives that are designed to address national Choosing Wisely campaign recommendations.

**Methods:**

Semi-structured interviews were conducted with individuals implementing Choosing Wisely Canada recommendations in healthcare settings in four provinces. The interview guide was developed using concepts from the literature and the Implementation Process Model (IPM) as a framework. All interviews were conducted virtually, recorded, and transcribed verbatim. Data were analysed using thematic analysis.

**Findings:**

Seventeen Choosing Wisely team members were interviewed. Participants identified numerous provider factors, most notably habit, which sustain LVC. Contrary to reporting in recent studies, the majority of LVC in the sample was not ‘patient facing’; therefore, patients were not a significant driver for the LVC, nor a barrier to reducing it. Participants detailed aspects of the magnitude of the problems of LVC, providing insight into the complexities and nuances of harm, resources and prevalence. Harm from potential or common infections, reactions, or overtreatment was viewed as the most significant types of harm. Unique factors influencing the processes of de-implementation reported were: influence of Choosing Wisely campaigns, availability of data, lack of targets and hard-coded interventions.

**Conclusions:**

This study explicates factors ranging from those which impact the maintenance of LVC to factors that impact the success of de-implementation interventions intended to reduce them. The findings draw attention to the significance of unintentional factors, highlight the importance of understanding the impact of harm and resources to reduce LVC and illuminate the overstated impact of patients in de-implementation literature. These findings illustrate the complexities of de-implementation.

**Supplementary Information:**

The online version contains supplementary material available at 10.1186/s12913-022-07485-6.

## Background

De-implementation - reducing or stopping the use of a health service or practice provided to patients by health care practitioners and health care delivery systems [1] - is an emerging field of study in healthcare. The reduction of LVC is necessary to ensure patient safety and satisfaction, reduce costs and develop a sustainable healthcare system [2]. It is estimated that 30% of current healthcare dollars are spent on unnecessary, wasteful, or harmful testing, procedures, and medications [3]. In recent years, several initiatives have been created, such as the international Choosing Wisely campaigns, which seek to identify and address the prevalence of low-value healthcare practices. Canadian hospitals and primary care providers are increasingly including Choosing Wisely Canada recommendations in their strategic priorities and are implementing numerous initiatives to reduce LVC.

Researchers have begun to explicate the complexities of de-implementation and draw attention to the distinction between factors that impact the use of LVC and factors that impact the processes of de-implementation [4, 5]. This distinction is important to understanding the root causes of LVC and also the barriers and facilitators to reducing inappropriate care [5–8]. Researchers have noted that patient factors are significant to the maintenance of LVC as well as cultural and historical factors [8–10]. The goals of de-implementing LVC are removing or reducing patient harm, maximizing efficient use of resources and improving population health [4, 11, 12].

There has been increasing exploration into the use of theory broadly in de-implementation efforts [4, 13]. Recent reviews have reported that de-implementation efforts have increasingly utilized classic theories, determinant frameworks, and implementation theories to support efforts to reduce LVC [10, 14]. Research is inconclusive on the value of implementation science knowledge for understanding de-implementation [4, 7, 8, 15–17]. A recent scoping review used an implementation science framework to understand determinants of de-implementation [5]. The authors found that while many determinants aligned, the importance of some factors varied and several unique determinants for de-implementation were identified. Similarly, Norton and Chambers [18] noted that like implementation, the practice, provider, patient, and setting are key aspects of the process, but for de-implementation the impact of factors at each of these levels may be different. Numerous provider factors have been identified as unique to de-implementation, such as the desire to meet patient requests, habit, fear of malpractice, fear of missing a diagnosis, and cognitive dissonance [1, 4]. Several authors have reported that patient barriers also impact de-implementation processes [5, 8, 10]. Prusaczyk, Swindle and Curran [8] noted that patient factors are more significant with certain LVC, particularly prescribing practices.

Increasingly, researchers and providers are implementing interventions to reduce LVC; these include varied intervention strategies such as education, audit, and feedback and system redesign [19]. As Choosing Wisely campaigns mature there is a call to move past education and awareness building, and produce empirical work focusing on the development and testing of interventions to de-implement LVC with measurable clinical and process outcomes [7, 20, 21].

To date, de-implementation research has focused primarily on identifying the problem of LVC. A more in-depth and nuanced understanding of the context in which LVC is practiced and in which de-implementation occurs can increase the effectiveness of efforts to reduce harm to patients and improve the use of resources, ultimately improving the overall health of populations [4, 18, 22]. In addition, there has been a call for qualitative research to explore the complexities of de-implementation and how determinants differ depending on the type of LVC [1, 5]. Building on previous work [14, 23] this study aims to understand, (i) factors that sustain LVC, (ii) the magnitude of the problem (LVC), and (iii) factors that impact de-implementation processes. These aims are accomplished through an exploration of Choosing Wisely Interventions implemented in hospital and primary care settings.

## Methods

### Study design

We used semi-structured interviews to address the study objectives and gain insight into the factors influencing the use of LVC, the magnitude of the problem of LVC, and unique aspects of de-implementation processes. A qualitative methodology is an ideal approach to explore perceptions and experiences of a phenomenon [24]. The Standards for Reporting Qualitative Research (SRQR) were used to guide the reporting of this study (See Additional file 1). This study has received ethics approval from the Office of Research Ethics at the University of Toronto (Protocol # 00038952) and the Office of Research Ethics at North York General Hospital (Protocol #20-0019).

### Study setting

Interventions related to Choosing Wisely Canada recommendations in hospital and primary care settings in four Canadian provinces.

### Study participants & recruitment

We used a purposive recruitment strategy. The inclusion criteria were: team lead or member that implemented an active intervention to address a Choosing Wisely Canada recommendation in the last five years. Our goal was to interview 15-20 participants based on feasibility and recommendations from the literature that qualitative studies typically reach data saturation after twelve interviews [25]. Participants were recruited through two methods. Initially, participants were recruited by a purposive sampling strategy through their participation in a hospital Choosing Wisely Canada Committee. Potential participants were contacted via email and were sent an email invitation that contained a short description of the participation requirements, expectations of participants, and a brief recruitment survey. One-third of participants (*n* = 6) were recruited through the initial strategy. Due to the impact of the Covid-19 pandemic on the availability of potential participants, we expanded the recruitment strategy to identify additional potential participants from individuals who participated in the Choosing Wisely Canada Annual National Meetings in 2018 & 2019. These potential participants were invited to participate in the study via their publicly available email addresses. All interested participants responded and provided high-level details about the Choosing Wisely Canada intervention they were involved in and their availability through the recruitment survey. Eleven participants were recruited through the second recruitment strategy. In total, 38 email invitations were sent to potential participants. Our goal was to obtain a diverse sample across hospital and primary care, geographic locations and targeted LVC. From the respondents, those who met the inclusion criteria were selected for an interview.

### Data collection

Interviews were selected as the ideal data collection method as they facilitate the exploration of context and the complex interplay between individuals, processes, and structures [26]. The interview guide was developed using concepts from the literature, the Framework for de-implementation in cancer care delivery [1] and the Implementation Process Model (IPM) [23]. In their conceptual framework, Norton, Chambers and Kramer [1] detail numerous factors in the Continuum of Factors Influencing De-Implementation Process, we explore the subfactors of harm, prevalence, and resources from the factor the *Magnitude of the Problem* of LVC. The IPM details key elements in the intervention implementation process, and questions were developed regarding the pre-implementation (planning) phase, implementation phase, monitoring, and evaluation. Interviews concluded with questions about participants’ experiences with de-implementation and how it differs from implementation efforts. In addition, the guide gathered data on pre-intervention rates, targets, intervention strategies, and outcomes. The interview guide was iteratively developed by the research team and finalized at 15 questions (see Additional file 2).

Interviews were approximately 1 h in length led by an experienced facilitator (GP) who had no affiliation or existing relationship with eligible participants. Due to the in-person meetings restrictions imposed by the Covid-19 pandemic, all interviews were conducted virtually via video conferencing. We obtained the participant’s written consent to participate, via email, in advance of the interview. Participants were sent the interview questions in advance of the interviews. Verbal consent for the interview to be video recorded was obtained at the beginning of the interview. Interviews were conducted between August and October 2020 and recruitment continued until no new insights emerged. Participants received $100.00 gift cards as compensation for their time.

### Data analysis

The transcripts were transcribed verbatim and analyzed using thematic analysis [27]. Initially during the familiarization phase, one researcher (GP) read and coded the transcripts and identified initial a priori codes, identified through the literature review, and used to create the interview questions, and new codes which emerged from the data. To ensure inter-rater reliability, three research team members (NS, MK, KB) independently coded a sample of interview transcripts, which were compared against the first team member’s coding of the transcripts. Discrepancies were resolved through consultation with the team. The team developed and refined the codebook iteratively by re-coding and refining a priori and emerging themes. The transcripts and codes were entered into NVivo 10 software, a qualitative analysis package (QSR International, 2020). The number of instances was tabulated to confirm dominant themes. Code saturation was reached when no new codes were identified across all transcripts.

## Findings

### Sample characteristics

Thematic saturation was reached with 17 interviews. The characteristics of interventions, participants and LVC are in Table 1. The participants represented Choosing Wisely Canada implementation efforts in four provinces across Canada from hospital (*n* = 15) and primary care (*n* = 2) settings and addressed a variety of LVC (see Table 1).

**Table 1:** Sample characteristics

			*(n=17)*
*Location*	Ontario		12
	Newfoundland		3
	Saskatchewan		1
	Nova Scotia		1
*Setting*	Hospital		15
	Primary Care		2
*Role in CWC Intervention*	Team Lead		12
	Physician Champion		2
	Team Member		3
*Low-value care*	Prescribing	Opioids	3
		Antibiotics	2
		PPIs	1
	Laboratory testing		5
	Pre-Operative testing		2
	Blood transfusions		2
	Imaging		1
	Catheter use		1

### Key findings

**Fig. 1:**
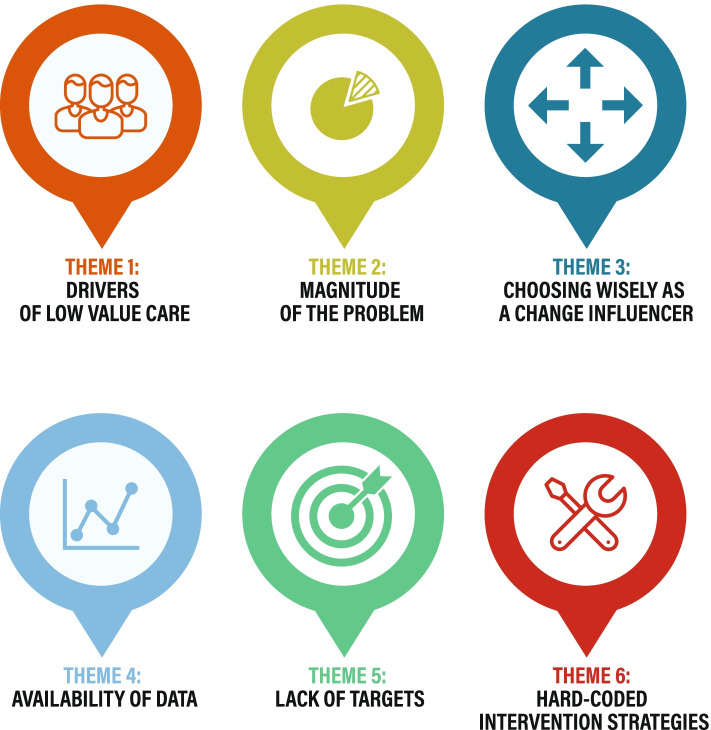
LVC & de-implementation: key themes

Our analysis of the data identified six major themes with subthemes relating to the research questions: drivers of LVC; the magnitude of the problem of LVC; and unique influences on de-implementation processes (see Fig. 1.).

### Theme 1: drivers of LVC

The first theme describes participant’s perspectives on the drivers of LVC. The majority of participants provided perspectives on factors deemed significant to the drivers of the targeted LVC. Participants noted the importance of understanding why the practice had reached inappropriate levels and also understanding what factors were sustaining the practice to better develop and implement interventions to reduce the LVC. Provider factors were prominently discussed, while patients were not identified as significant factors for the majority of LVC.

#### Provider factors

The most common factors discussed related to providers, such as habituation, years of practice, training, and a belief that ‘more is better’. A low-value practice being done through habit was identified as a significant factor in the sustainment of the practice. Some participants noted how habit extended to the institution and society.

*I think a lot of the resistance was just related to people having their own style of practice, they’ve been doing [it] a certain way for so many years … probably the strongest resistance to this project was the force of habit. [P10]*

In addition to the practice being ingrained for the provider, the practice being perceived as ‘status quo’ and supported by the system and patient expectations were identified as factors:

*…the biggest barrier is culture, that this is the way you’ve practiced for a long time and this is the way that the population is expecting practice. It’s the combination of demand from the population and a system where it’s easier to meet the demand than push back on the demand. [P12]*

Many participants pointed to years of practice or training, either medical school or in the institution, as additional ways the LVC was sustained:

*There was a lot of people who were very resistant as well. So, there’s quite a bit who had got quite defensive and put their backs up. And usually, it was kind of the older physicians that I would say they were kind of set in their ways. [P17]*

Participants noted that providers endeavour to provide optimal care for their patients and this can often motivate more care than is necessary. Participants reported that providers are motived to continue LVC because of concerns about misdiagnosis, reputation and a desire to meet expectations.

*There was a culture of wanting to be very thorough and doing a lot of testing in order to demonstrate that you were really keeping an eye on things and being expansive in your differential diagnosis. So, there’s that aspect of wanting to impress. [P03]*

#### Patient factors

The majority of the LVC in this study were not patient-facing, meaning that patients demands or expectations or clinician perceptions of these patient-drivers of LVC, did not exert significant influence on the sustainment of the practice. Duplicative lab testing, unnecessary blood transfusion volumes, indwelling catheters, and antibiotics in the ICU are some of the practices which were not patient-facing and therefore were not influenced by patient expectations and demands.

### Theme 2: the magnitude of the problem of LVC

The second theme describes the significance of understanding the magnitude of the problem of LVC through the concepts of harm, resources and prevalence. In the analysis, we identified that harm occurs on multiple levels. Participants discussed the significance of harm and how the recognition of this harm motivated the change. Resources were also a significant factor that motivated the decision to reduce the LVC. These Choosing Wisely Canada implementation efforts took many aspects of resources – from time to human resources, to financial – into consideration when deciding to reduce the LVC. Finally, the prevalence was an important yet complex factor. Project teams recognized that the level of the LVC was inappropriate but were often challenged to identify specifically how prevalent the practice was.

#### Harm

Almost all of the participants discussed the harm from the LVC as an important factor motivating efforts to reduce the practice. Harm can come from the actual performance of the LVC, from the potential or common downstream effects of the LVC, from longer-term effects on patients, or from downstream harm to population health. The physical harm to the patient from doing the actual procedure or practice was predominantly reported as the least significant harm. This direct harm, e.g., an additional blood draw or excessive radiation, was deemed minimal compared to other types of harm from the LVC:

*…although x-rays have low exposure, the dose [of radiation from] a rib x-ray which requires several views of both ribs, definitely is a concern for harm. That’s the main, I would say, patient harm. [P02]*

Potential and common physical harm to the patient resulting from the LVC, such as infections, antimicrobial resistance or overtreatment were commonly discussed:

*…causing an infection and that infection can spread and it can infect your orthopedic implant and that can be a pretty horrendous complication if that happens. But even short of that, just having a UTI is a problem. Patients can get disseminated sepsis from that. So that’s a big problem and then just delirium as well, from having the catheter, from having a UTI. [P16]*

Over-testing and overtreatment as a result of the LVC was also discussed:

*When urine cultures are ordered incorrectly, they [can] lead to antimicrobial overuse… because we’re doing the testing inappropriately, you’re going to get a 15 to 50% positive rate of positive bacteria, which will lead to treatment and that has no benefit. [P02]*

Addiction, overdoses and infections were discussed as potential or common downstream harm to patients:

*There is still a significant portion of patients who overdose on medications like Hydromorphone or prescribed Fentanyl patches or Morphine for that matter. So, that’s still a significant problem. [P04]*

The impact of the LVC on population health was an important consideration for tackling the issue at most participants’ institutions:

*…if we start overusing antibiotics the bacteria become resistance, then you’re going to have troubles down the road where people actually need these bacteria antibiotics, and the antibiotics are not going to work. They’re not going to be lifesaving. [P17]*

#### Resources

The resource aspect of LVC was discussed by all participants. Resources belong to a broad category that encompasses patient and provider time, medical equipment, and supplies. A few participants stated that for their Choosing Wisely Canada effort, harm was not the primary driver, but rather wasted resources. One participant detailed the multiple aspects of wasted resources presented by the LVC:

*…you’re using more [blood] products and the products are valuable products [and] are not always available. You’re using more lab time in doing the cross match and the issuing of the units, you are using more nursing time spending the time transfusing the unit. You are using more tubing system because each system has to have tube as well and you’re using the patient’s time, sitting there and receiving the transfusion. Giving an unneeded intervention is a huge waste of resources. [P15]*

The waste of resources, not physical harm to patients, was a significant motivator for some of the initiatives:

*…it would be more harm in the sense of wasted resources on the system, more than actual patient harm I would say. Because it’s really hard to see, the idea of unnecessary testing is an important one, but because there’s very little patient harm coming from it, it’s hard to sort of make an argument for it. [P11]*

*…one of our challenges in MRI is we have a wait list. And so those cases should be for indications that require MRI because there’s no other diagnostic. So, we really just don’t want to be using up the time on stuff that’s not going to change management [of care]. [P09]*

Some participants discussed not only the immediate resources wasted, but also downstream waste produced and the burden to the healthcare system:

*“We have a very high opioid overdose rate, [x] times the rest of the province and then our hospitalization and emergency department visits were also about [x] times [the] rest of the province. So, these patients, they certainly can take up a lot of resources. These patients go to walk in clinics, the emergency department multiple times a month. They’re admitted for months at a time with infectious complications from injection IV drug use, endocarditis, all these sorts of things. So, even the prevention of one or two of these patients I think has a significant impact to resources of the healthcare system. [P04]*

The volume of tests and procedures was taken into account when assessing the impact on resources:

*…the cost of doing the test is quite low, maybe it’s about two bucks a test. But the quantity of testing is so high that it translates into a substantial amount of spending. [P11]*

#### Prevalence

In the context of the magnitude of the problem, the prevalence of the LVC was discussed by the majority of participants. Often participants knew how often the practice was being performed, but not how often the practice was being performed inappropriately. Some project teams collected data on practice rates pre-intervention while others started the intervention, with the soft goal of reducing inappropriate practice, without baseline data.

*So, I would say about three-quarters of them were getting blood work. About two-thirds were getting ECGs and about 25% were getting chest x-rays. And these are numbers that could all essentially go to zero because we’re already talking about the population that doesn’t need them. We’re talking about low-risk patients getting low-risk surgery. [P08]*

### Unique influences on de-implementation processes

Four major themes were identified that provide insights on participant perspectives on the unique influences on de-implementation processes. The interview guide walked participants through the implementation process of the interventions to reduce the LVC. Participants discussed various aspects of the implementation process, from planning to implementation, to monitoring and evaluation. Participants were asked if they used theory to develop their strategy, identify barriers and facilitators, or select intervention strategies. While a number of initiatives were quality improvement projects, none of the participants reported using theory to inform or guide the initiative. The themes described in this section highlight some unique aspects of de-implementation processes.

### Theme 3: Choosing Wisely as a change influencer

Participants discussed many aspects of how the Choosing Wisely Canada campaign supported de-implementation efforts. They discussed that Choosing Wisely Canada is respected, well known in the medical community, and had done much to increase awareness about LVC and the benefits of reducing it. The impact of Choosing Wisely campaigns was also perceived to generally influence culture and support providers to question existing practices:

*Choosing Wisely was really the catalyst of de-implementation. It was an awareness. It was a lot of education here. [P05]*

### Theme 4: availability of data

Participants discussed the challenges around data availability and how these challenges impacted de-implementation efforts. Data acted as both a facilitator and a barrier for the included interventions.

*You need to look and analyze what’s driving that change. Sometimes it’s practice. Sometimes it’s a knowledge gap sometimes, it’s an evolution of care that’s happened over time. We’re fortunate in that we can pull data very easily around what volumes are we looking at? Where are those volumes coming from? And then from that you can make some inference about why it may be high in one area versus the other and where can we tackle it? When we look at it, we’re able to not only look at volumes but also the source of those orders. [P06]*

*I would say this is a system level barrier in general for all quality improvement – data - and being able to track this stuff. It’s really hard… the amount of time that went into getting all that data by this painful chart review because we don’t have it at our fingertips, just because of how the systems are set up…at the end of the day then one of your limitations is the quality of that data … So, having metrics actually available for these things that are important would be a huge benefit and is a barrier always to doing this kind of work. [P16]*

### Theme 5: lack of targets

The lack of targets, and the difficulty inherent in establishing them, were identified by participants. These projects were often at the forefront of this type of practice change and project teams often had little data on both what was deemed appropriate for the practice, and what level of practice at their particular institution was inappropriate:

*…but there’s so many interventions that we overdo that there’s no clear recommendation on what the target should be. So, I think it’s actually more interesting that we didn’t use a target. To show that we were still able to make a reduction without having a true target and that would be maybe more harmful than useful. [P02]*

*Well, the goals were obviously to reduce the amount of volume of antibiotics or the rate of antibiotics in the province. The issue with that is, we never really had a goal because we couldn’t really identify appropriateness. [P17]*

*…we didn’t really know what the problem was; there’s no agreed upon number. No one knows what proportions of patients actually need to be seen or what proportion of those patients actually needs a test. Those sorts of targets don’t exist. [P11]*

### Theme 6: hard-coded intervention strategies

Approximately a third of participants discussed the differences in hard-coded versus soft interventions. Hard-coded interventions are those typically built into systems or technology, where soft interventions, such as education or guidelines, rely on individuals to enact them. Participants discussed how hard-coded interventions were often beneficial but were sometimes associated with unique challenges relating to LVC. For a few of the practices, root cause analysis identified that LVC was prevalent because it was part of an existing order set or directive, not because the practice was being sustained intentionally.

*…it was a very sustainable change because it was hardwired into practice and workflow as opposed to sometimes other things where you’re more reliant on people to remember or remain committed. [P06]*

*…so interestingly and we didn’t realize this, the vast majority of two-unit [blood] transfusions were ordered on admission as a standing order. So, it was not a deliberate choice. At the time of the transfusion, it was a pre-standing order that the physician had just entered and left there. [P03]*

## Discussion

**Fig. 2:**
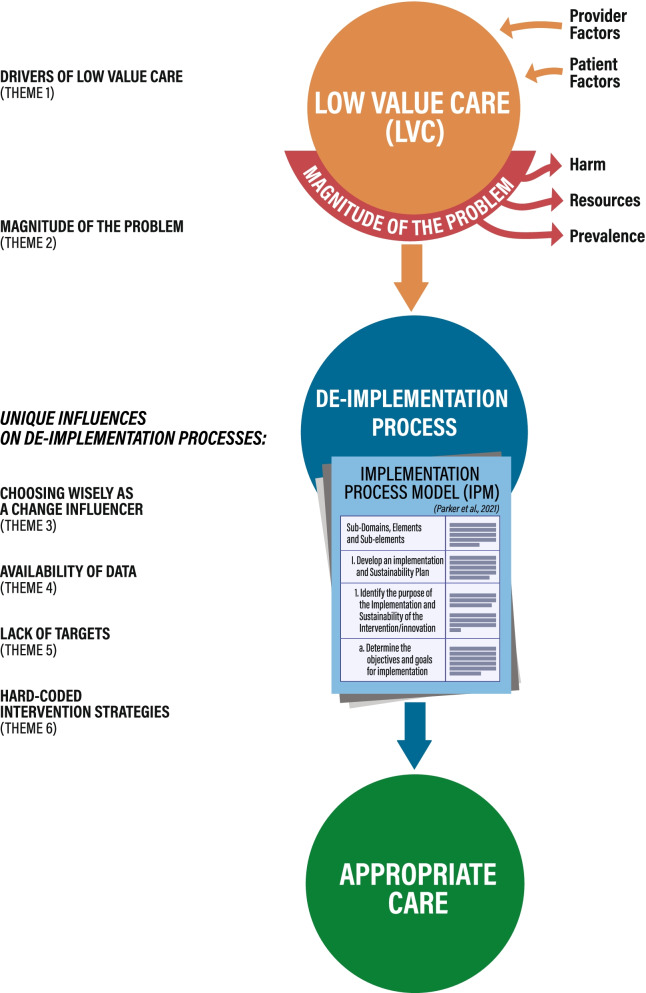
LVC & de-implementation: themes, sub-themes and relationships

The participants of this study provided valuable insights into de-implementation processes. The eight different low-value practices in the sample represent a diverse cross-section of common LVC with the majority of these practices reflected in the Choosing Wisely international Top 10 Recommendations List [7]. The figure below (Fig. 2) illustrates the relationships between the themes and subthemes discussed in this study.

To our knowledge, this is the first study to explicate the magnitude of the problem of LVC through the concepts of harm, resources, and prevalence. Discussing these aspects of LVC allowed participants to provide details about the complexities and nuances of the negative impact of LVC. Harm reduction was a significant motivator to reduce the LVC. The findings demonstrate that harm is a nuanced concept and encompasses immediate harm to the patient, potential or common downstream impacts to the patient, longer-term harm to the patient, and harm to population health. Participants viewed harm from potential or common infections, reactions, or overtreatment as the most significant type of harm. Numerous participants spoke of the broad implications, to society and future generations, of inappropriate prescribing practices and the increased use of healthcare services that result from LVC. Aligning with the literature, participants reported that providers, organizations, and patients were often unaware of the significance of the harms presented by the LVC [28, 29]. This lack of awareness identifies a gap to be addressed by de-implementation initiatives where fulsome analysis of the magnitude of the problem of LVC during the planning phase of a given the project could identify where attention should be focused and determine which interventions are best suited to address the specific harms presented by the LVC.

Waste in healthcare is recognized as a significant issue [1, 30]. Resources can be categorized as wasted supplies and equipment, in addition to wasted time on the part of patients and providers. While costs of unnecessary medical equipment and supplies can easily be quantified, the downstream savings of not overtreating or over-testing is less easily calculated. Time and human resources waste are also challenging to calculate. Addressing costs and financial savings as an aspect of reducing LVC has been reported as a contentious and difficult aspect to address [16, 31]. Choosing Wisely Canada and campaigns internationally have purposely steered away from the financial aspect of reducing LVC, particularly in an effort to mitigate concerns about rationing care or reducing physician income [32]. In addition, different jurisdictions, e.g., Canada and United States, have very different financial structures for healthcare. In Canada, the financial aspect of the healthcare system has less of a direct impact on patient finances. These findings suggest that direct costs are only a portion of the waste that occurs with LVC. It may be more palatable, and just as valid, to address wasted equipment, supplies, and time than costs and financial savings when planning to reduce LVC.

The prevalence of the LVC was a novel concept explored by this study. Our findings provide insight into how participants assessed the prevalence of the LVC and how they determined if the practice was inappropriate and required remediation. Some participants collected baseline data to try to understand the current practice levels, but more often, participants stated that the project team knew that a certain level of practice was inappropriate but did not have data to substantiate it. This issue is unique to de-implementation. As the field matures more research will be published on measures of appropriateness that can guide future de-implementation initiatives.

Provider factors, such as habituation, training, years of practice, and belief that ‘more is better’ were identified as having a significant impact on the maintenance of LVC. Overwhelmingly participants recognized the challenges inherent in behaviour change. The majority of participants noted that the LVC had become a habit for providers and that habituation contributed to the level of overuse that existed prior to the intervention. Research has reported that a significant proportion of a healthcare provider’s daily work is habitual rather than intentional and that routines do not require active decision-making [33, 34]. Participants often reported that providers were not aware of updated evidence regarding the LVC which aligns with recent research [9]. The role of habit in sustaining LVC has not yet been explored in the literature and understanding the factors that contribute, the processes and mechanisms involved in habit forming and breaking are critical to de-implementation efforts.

Another significant factor that sustained the LVC was the belief that ‘more is better’. Participants noted that providers often wanted to “err on the side of caution” and order more tests than necessary or prescribe more medication. Participants reported that providers worried about the opinions of colleagues and that there would be an expectation that they would not be meeting if they did not order tests or provide medications. Research has reported that perceived pressures from other physicians or the healthcare system significantly impact LVC use [9, 35]. In a recent study of physicians’ experiences with Choosing Wisely, the authors reported that “all physician participants reported that patients often enter the clinical encounter with information and expectations about care” ([32]:3). This statement gives the impression that all LVC has a significant patient facing component. In our sample, patient factors were not significant in sustaining the LVC. Our findings are also contrary to much of the recent literature on the topic [5, 8–10, 36]. The difference in our findings can be explained by the fact that the majority of our sample (12 of 17) of low-value practices were not ‘patient-facing’. While some LVC, such as inappropriate antibiotic or diagnostic imaging use can be impacted by patient factors, these low-value practices – reducing inappropriate blood use, reducing duplicative lab testing, antibiotic use in the ICU, indwelling catheters – are typically not impacted by patient requests or expectations. This is an important finding which highlights that a significant proportion of LVC is not patient-facing. The myriad other factors driving LVC merit recognition by researchers and be accurately identified and targeted to support successful de-implementation efforts.

Participants reported numerous factors which supported or hindered the de-implementation processes. Many of these factors were similar to those reported in the implementation literature [5, 8], but the impact of factors such as the influence of Choosing Wisely campaigns, availability of data, lack of targets, and hard-coded interventions are unique to de-implementation. Overwhelmingly, participants reported on the impact of Choosing Wisely on education, awareness, and support for efforts to reduce LVC. Participants discussed how Choosing Wisely campaigns have acted as a ‘catalyst’ for change and provided ‘validation’ for these initiatives. These perspectives align with findings in the literature which report that Choosing Wisely campaigns have been recognized as providing validation and legitimation to efforts to reduce LVC, particularly with patients and the public [37].

For project teams executing Choosing Wisely Canada related implementation efforts, data could act as a barrier or a facilitator. Participants noted that, when available, data was a significant facilitator, enabling teams to demonstrate the amount of inappropriate use, the root cause of the overuse, or disparity in use between departments. Other participants noted that a lack of data or difficulty accessing or obtaining data was a significant barrier to their initiative. The challenges that data availability and quality of data for de-implementation efforts have been recognized in the literature [31, 38]. Unique to de-implementation and discussed by participants was the fact that the level of “inappropriateness” of the practice was not often known and therefore targets for reduction could not be established. This aspect of de-implementation has not yet been explored in empirical studies. Camerini et al. [30] noted that the idea of appropriateness is conceptually challenging for providers as the appropriateness of a given practice is connected to both cultural evolution and the production of new data. The participants discussed appropriateness and noted that it was not only conceptually, but practically challenging. For some LVC, there is scarce empirical evidence on what is an appropriate amount for the practice. Participants discussed this unique challenge when planning for and executing the intervention. Participants stated that they knew that a percent of the practice was inappropriate but did not know what the appropriate level should be. Often participants referenced similar published studies to provide guidance but pointed out the importance of understanding the level of inappropriateness in their own organization. The lack of this data was often dealt with by not having targets for reduction or deciding that any reduction was appropriate for the first round of the intervention. Not having targets for reduction also complicated monitoring and evaluation processes for these initiatives. This aspect of de-implementation was new for many participants. The challenge of defining and measuring optimal outcomes, [7, 18] is recognized by de-implementation researchers. In addition, the authors have noted that the absence of comparative data makes it difficult to determine whether the reduction in practice is clinically useful [30].

For participants of the study, hard-coded interventions were seen as favourable and effective to make sustainable change. Modifying order sets has been recognized as an effective strategy to reduce inappropriate practices such as laboratory testing [1]. While hard-coded interventions were seen as effective to reduce the burden on providers, a few participants illustrated the negative side of these interventions – LVC can be part of an order set and forgotten about and therefore inadvertently continued when it is no longer appropriate. Aligning with these findings, the pitfalls of order sets and their passive maintenance of LVC have also been noted in the literature [19].

## Implications for future research

To our knowledge, this is the first study to explicate the magnitude of the problem of LVC through the concepts of harm, resources and prevalence. Efforts to reduce LVC should include a detailed analysis of the magnitude of the problem, including a thorough understanding of the complexities of harm, resources and prevalence. More research is needed to identify the distinctions between LVC driven by patient factors and LVC which is not impacted by patient demand and expectations. The findings of this study further highlight the need to understand the impact of data availability and targets on de-implementation efforts. Empirical studies are needed which explore LVC driven by passive provider factors, such as habit and years of experience.

## Strengths and limitations

This study has numerous strengths. Using a qualitative approach provided a deeper understanding of the complexities and nuances of LVC and de-implementation processes. In addition, using a theory-based approach to data collection and analysis added rigour to the study. The study had a few limitations, most notably the impact of the Covid-19 pandemic on participant availability and data collection. Healthcare providers have been called upon to unprecedented lengths during the pandemic and understandably participation in research was not a priority. Even under these circumstances, many individuals participated, providing insightful and meaningful data, during these challenging times. While the research team is very grateful for all who participated, it should be noted that the sample diversity may not be as rigorous as it would have been pre-pandemic. While the sample diversity may be a limitation, saturation was reached for all themes. The fact that all interviews were conducted virtually may also be a limitation of this study. As is common with qualitative research, participants may have given socially desirable answers to some interview questions.

## Conclusion

The findings of this study provide insight into factors that sustain LVC, the magnitude of the problem of LVC, and unique aspects of de-implementation processes. De-implementation researchers should explore passive, unintentional factors which drive LVC, drivers for LVC that are not patient-facing, and understand the impact of harm, resources and prevalence on efforts to reduce LVC.

## Supplementary Information

**Additional file 1.** SRQR Checklist.

**Additional file 2.** Interview Guide.

## Data Availability

The datasets supporting the conclusions of this article are included within the article and its additional files.

## Abbreviations

LVC: Low-value care
IPM: Implementation Process Model

## References

[1] Norton WE, Chambers DA, Kramer BS (2019). Conceptualizing de-implementation in cancer care delivery. J Clin Oncol Off J Am Soc Clin Oncol.

[2] Paprica PA, Culyer AJ, Elshaug AG, Peffer J, Sandoval GA (2015). From talk to action: policy stakeholders, appropriateness, and selective disinvestment. Int J Technol Assess Health Care.

[3] Health Quality Ontario (2017). Spotlight on leaders of change. Implementing Choosing Wisely Canada recommendations in Ontario to improve quality of care.

[4] Grimshaw JM, Patey AM, Kirkham KR, Hall A, Dowling SK, Rodondi N, Ellen M, Kool T, van Dulmen SA, Kerr EA, Linklater S (2020). De-implementing wisely: developing the evidence base to reduce low-value care. BMJ Qual Saf.

[5] Augustsson H, Ingvarsson S, Nilsen P, von Thiele Schwarz U, Muli I, Dervish J, Hasson H (2021). Determinants for the use and de-implementation of low-value care in health care: a scoping review. Implement Sci Commun.

[6] Davidson KW, Ye S, Mensah GA (2017). Commentary: De-implementation science: a virtuous cycle of ceasing and desisting low-value care before implementing new high value care. Ethn Dis.

[7] Born KB, Levinson W (2019). Choosing Wisely campaigns globally: a shared approach to tackling the problem of overuse in healthcare. J Gen Family Med.

[8] Prusaczyk B, Swindle T, Curran G (2020). Defining and conceptualizing outcomes for de-implementation: key distinctions from implementation outcomes. Implement Sci Commun.

[9] Ingvarsson S, Augustsson H, Hasson H, Nilsen P, von Thiele Schwarz U, von Knorring M (2020). Why do they do it? A grounded theory study of the use of low-value care among primary health care physicians. Implement Sci.

[10] Nilsen P, Ingvarsson S, Hasson H, von Thiele Schwarz U, Augustsson H (2020). Theories, models, and frameworks for de-implementation of low-value care: a scoping review of the literature. Implement Res Pract.

[11] Marcotte LM, Zech JM, Liao JM (2021). Key features underlying low-value care recommendations. Am J Med Qual..

[12] Kim DD, Do LA, Daly AT, Wong JB, Chambers JD, Ollendorf DA (2021). An evidence review of low-value care recommendations: inconsistency and lack of economic evidence considered. J Gen Intern Med..

[13] Patey A, Grimshaw J, Francis J (2021). Changing behaviour, ‘more or less’: do implementation and de-implementation interventions include different behaviour change techniques?. Implement Sci.

[14] Parker G, Shahid N, Rappon T, Kastner M, Born K, Berta W. Using theories and frameworks to understand how to reduce low value healthcare: a scoping review. Implement Sci. 2022. 10.1186/s13012-021-01177-1. 10.1186/s13012-021-01177-1 PMC8772067 35057832

[15] Niven DJ, Mrklas KJ, Holodinsky JK, Straus SE, Hemmelgarn BR, Jeffs LP, Stelfox HT (2015). Towards understanding the de-adoption of low-value clinical practices: a scoping review. BMC Med.

[16] Montini T, Graham ID (2015). “Entrenched practices and other biases”: unpacking the historical, economic, professional, and social resistance to de-implementation. Implement Sci.

[17] van Bodegom-Vos L, Davidoff F, Marang-van de Mheen PJ (2017). Implementation and de-implementation: two sides of the same coin?. BMJ Qual Saf.

[18] Norton WE, Chambers DA (2020). Unpacking the complexities of de-implementing inappropriate health interventions. Implement Sci.

[19] Born K, Kool T, Levinson W (2019). Reducing overuse in healthcare: advancing choosing wisely. BMJ.

[20] Prasad V, Ioannidis JP (2014). Evidence-based de-implementation for contradicted, unproven, and aspiring healthcare practices. Implement Sci..

[21] Gnjidic D, Elshaug AG (2015). De-adoption and its 43 related terms: harmonizing low-value care terminology. BMC Med.

[22] Parker G, Rappon T, Berta W (2019). Active change interventions to de-implement low-value healthcare practices: a scoping review protocol. BMJ Open.

[23] Parker G, Kastner M, Born K, Berta W (2021). Development of an implementation process model: a Delphi study. BMC Health Serv Res..

[24] Sandelowski M, Barroso J (2003). Writing the proposal for a qualitative research methodology project. Qual Health Res.

[25] Guest G, Bunce A, Johnson L (2006). How many interviews are enough? An experiment with data saturation and variability. Field Methods.

[26] Creswell JW, Klassen AC, Plano Clark VL, Smith KC, Working Group Assistance (2011). Best practices for mixed methods research in the health sciences.

[27] Braun V, Clarke V (2006). Using thematic analysis in psychology. Qual Res Psychol.

[28] Morgan DJ, Brownlee S, Leppin AL (2015). Setting a research agenda for medical overuse. BMJ.

[29] Helfrich CD, Rose AJ, Hartmann CW, van Bodegom-Vos L, Graham ID, Wood SJ, Majerczyk BR, Good CB, Pogach LM, Ball SL, Au DH (2018). How the dual process model of human cognition can inform efforts to de-implement ineffective and harmful clinical practices: a preliminary model of unlearning and substitution. J Eval Clin Pract.

[30] Camerini F, Fabris E, Sinagra G (2019). Appropriateness, inappropriateness and waste of resources: unfulfilled expectations?. Eur J Intern Med.

[31] Kherad O, Peiffer-Smadja N, Karlafti L, Lember M, Van Aerde N, Gunnarsson O (2020). The challenge of implementing less is more medicine: a European perspective. Eur J Intern Med..

[32] Embrett M, Randall GE (2018). Physician perspectives on Choosing Wisely Canada as an approach to reduce unnecessary medical care: a qualitative study. Health Res Policy Syst.

[33] Nilsen P, Roback K, Broström A, Ellström PE (2012). Creatures of habit: accounting for the role of habit in implementation research on clinical behaviour change. Implement Sci.

[34] Potthoff S, Rasul O, Sniehotta FF, Marques M, Beyer F, Thomson R, Avery L, Presseau J (2019). The relationship between habit and healthcare professional behaviour in clinical practice: a systematic review and meta-analysis. Health Psychol Rev.

[35] Powell AA, Bloomfield HE, Burgess DJ, Wilt TJ, Partin MR (2013). A conceptual framework for understanding and reducing overuse by primary care providers. Med Care Res Rev.

[36] van Dulmen SA, Naaktgeboren CA, Heus P, Verkerk EW, Weenink J, Kool RB, Hooft L (2020). Barriers and facilitators to reduce low-value care: a qualitative evidence synthesis. BMJ Open.

[37] Rumball-Smith J, Shekelle PG, Bates DW (2017). Using the electronic health record to understand and minimize overuse. JAMA.

[38] Levinson W, Kallewaard M, Bhatia RS, Wolfson D, Shortt S, Kerr EA (2015). ‘Choosing Wisely’: a growing international campaign. BMJ Qual Saf..

