# Supplementary material for: Understanding low-value care and associated de-implementation processes: a qualitative study of Choosing Wisely Interventions across Canadian hospitals
Source: BMC Health Serv Res. 2022 Jan 21;22:92. doi: 10.1186/s12913-022-07485-6 (PMC8776509; doi:10.1186/s12913-022-07485-6)
Supplement: Supplementary file 2 — Additional file 2. Interview Guide. [file 12913_2022_7485_MOESM2_ESM.docx]

**LVC & De-implementation Study - Interview Questions**

1. **Intervention Characteristics**

*This information will be obtained from the documents that participants will send prior to the interviews. In the interview I will confirm details about the Choosing Wisely Intervention:*

- Target practice (e.g. reducing PPIs); Pre-intervention practice rates; Goal practice reduction rate/number (e.g. reduce PPI prescribing by 25%); Actual practice reduction rate/number (e.g. reduced PPI prescribing by 17%)

**Questions**

1. Describe the low-value practice that was being reduced.
2. Describe any cultural or historical significance of the practice (Prusaczyk et al., 2020)
3. Describe how the scope of the problem was assessed. For example, the harm, prevalence, and resources related to the low-value practice.
4. **Implementation Process**

*Describe the process when you implemented the X intervention to address the Y Choosing Wisely Recommendation.*

PRE-IMPLEMENTATION (Planning):

1. Describe the process regarding stakeholder involvement and engagement. What did the engagement look like? Frequency, dose?
   1. *Prompts:*
      1. *Did you engage stakeholders prior to beginning the implementation?*
      2. *Were stakeholders engaged throughout the intervention?*
      3. *Did you engage clinicians, champions, managers, front-line staff, QI specialist, senior leadership, policy makers, decision-makers, researchers, impacted staff, patients, care givers?*
      4. *Did you identify and clarify roles? Establish expectations?*
      5. *How did you determine communication mechanisms*
2. Did you develop an implementation plan *a priori*?
   - 1. *Did you determine the objectives and goals for the implementation?*
     2. *Did you identify the specific outcomes and outputs?*
3. Were any theories, models or frameworks used to guide implementation?
4. How did you select the most appropriate intervention?
   1. Did you assess fit and effectiveness of potential interventions prior to selection?
   2. Did you adapt or tailor the intervention pre-implementation?
   3. How was context incorporated in intervention selection?
5. Were there any processes to identify barriers and facilitators prior to intervention selection?
   1. *Prompts:*
      1. *Assess readiness to change – tools used?*
      2. *Patient level barriers?*
         1. *3 patient factors (fear and anxiety, inaccurate perceptions about health interventions and healthcare, lack of trust)* (Norton & Chambers, 2020)
      3. *Provider-level barriers?*
      4. *Technology/ system level barriers?*
      5. *Hospital level barriers?*
6. What outcomes or indicators did you select? Why?
7. Which of these outcomes did you measure?
   1. *Prompts: Clinical, Patient, Informal caregiver, volunteer, family member, Provider, Process or implementation, Service, Organizational and health care system outcomes, Economic*
8. How and when was sustainability considered in the implementation process?
9. Did you develop a monitoring plan *a priori*? Describe?
10. Did you develop an evaluation plan *a priori*? Describe?

IMPLEMENTATION:

1. Describe the Intervention strategy (e.g. education and audit and feedback) (including complexity factors: required additional staff, resources, time, acquisition of new skills, how long the inappropriate intervention has been in place, complex technical components, new practice contradicts in form function or philosophy the inappropriate intervention)
2. How closely did the actual implementation follow the planned implementation? If it differed, how did it differ?
3. Looking back, is there anything you did not address, plan for, or mitigate that you feel negatively impacted the implementation of the intervention?

POST-IMPLEMENTATION (Monitoring and Evaluation):

1. How closely did the monitoring and evaluation follow the plan? How did it differ?
2. Describe considerations of fidelity -was the intervention implemented consistently with the planned intervention?
3. Was the intervention changed or adapted? How?
4. Describe any barriers identified post-implementation.
5. **De-implementation**

**You very likely implemented before….**

1. Describe unique aspects of de-implementation identified during this initiative?
2. Describe unique barriers or facilitators identified?
